# Convenience-Oriented Dietary Behavioral Patterns Across BMI Classes in University Students: Associations with Overweight and Obesity Risk During the Transition to University Life

**DOI:** 10.3390/nu18142368

**Published:** 2026-07-20

**Authors:** Radu Dumitru Moleriu, Teodora Piroș, Lavinia Cristina Moleriu, Călin Muntean, Raluca Lupușoru, Anca Mihaela Dicu, Sebastian Ștefănigă, Ruxandra-Cristina Marin

**Affiliations:** 1 Departament III Functional Science, Discipline of Medical Informatics and Biostatistics, “Victor Babes” University of Medicine and Pharmacy, 300041 Timisoara, Romania; radu.moleriu@umft.ro (R.D.M.); teodora.piros@student.umft.ro (T.P.); raluca.lupusoru@umft.ro (R.L.); 2 Center for Modeling Biological Systems and Data Analysis, “Victor Babes” University of Medicine and Pharmacy, 300041 Timisoara, Romania; 3 Gastroenterology and Hepatology Clinic, County Emergency Hospital “Pius Brinzeu”, 300723 Timisoara, Romania; 4 Faculty of Food Engineering, Turism and Environmental Protection, Aurel Vlaicu” University of Arad, 310130 Arad, Romania; anca.dicu@uav.ro; 5 Faculty of Computer Science, West University of Timisoara, 300223 Timișoara, Romania; sebastian.stefaniga@e-uvt.ro; 6 Discipline of Pharmacology, Clinical Pharmacology and Pharmacotherapy, “Carol Davila” University of Medicine and Pharmacy, 050474 Bucharest, Romania; ruxandra.marin@umfcd.ro; 7 Doctoral School of Biological and Biomedical Sciences, University of Oradea, 410087 Oradea, Romania

**Keywords:** dietary patterns, eating behavior, overweight, obesity, university students, fast-food, dietary risk score, logistic regression, cross-sectional, Romania

## Abstract

**Background/Objectives**: Convenience-oriented dietary behaviors acquired during the transition to university life may be associated with overweight and obesity status in young adults. This study aimed to characterize the dietary behaviors of Romanian university students and to evaluate their associations with overweight and obesity. **Methods**: This cross-sectional study included 921 university students aged 18–24 years (60.6% female; 67.8% urban residents). Self-reported dietary variables included fast-food consumption, sweets intake, fruit and vegetable intake, water intake, and replacement of meals with desserts. BMI categories were classified according to WHO criteria. Associations were evaluated using chi-square tests, Cramer’s V, and multivariable logistic regression adjusted for sex, age group, and residence environment. A five-item Dietary Risk Score (DRS; range 0–5) was constructed as an exploratory composite indicator of cumulative dietary behavioral burden. Cluster analysis was additionally performed to identify dietary behavioral phenotypes. **Results**: A total of 24.1% of participants were classified as overweight/obese, including 19.4% overweight and 4.7% obesity. Frequent replacement of meals with desserts (aOR 7.20; 95% CI 4.26–12.17) and fast-food consumption ≥3 times/week (aOR 1.98; 95% CI 1.26–3.10) were independently associated with overweight/obesity after adjustment for demographic variables. Male sex, older age, and urban residence were also independently associated with overweight/obesity. Distinct dietary behavioral phenotypes were identified, including a convenience-oriented cluster characterized by higher fast-food and sweets intake and the highest prevalence of overweight/obesity. The DRS demonstrated a dose–response relationship with overweight/obesity prevalence, increasing from 19.8% at DRS = 0 to 50.0% at DRS = 4, with an adjusted OR of 1.43 per one-point increase (95% CI 1.23–1.66; *p* < 0.001). **Conclusions**: Frequent meal replacement with desserts, higher fast-food consumption, and greater cumulative dietary behavioral burden were associated with overweight and obesity status among Romanian university students. The findings support the relevance of evaluating cumulative dietary behavioral patterns in young adults; however, the cross-sectional design precludes conclusions regarding causality or temporal directionality.

## 1. Introduction

According to the World Health Organization, more than 2.5 billion adults worldwide were overweight in 2022, including approximately 890 million individuals living with obesity, while global obesity prevalence has more than doubled since 1990 [1]. Recent Global Burden of Disease forecasts estimate that, if current trends continue, approximately 3.80 billion adults worldwide will be living with overweight or obesity by 2050, representing more than half of the global adult population [2]. European epidemiological data similarly emphasize the magnitude of the problem. According to Eurostat statistics published in 2024, approximately 50.6% of adults in the European Union are currently overweight, while obesity prevalence continues to increase among younger age groups [3]. In the WHO European Region, overweight and obesity currently affect almost 60% of adults and nearly one in three school-aged children, highlighting the persistence of obesogenic environments across the life course and the growing importance of early preventive interventions [4]. Thus, excess body weight has become one of the most significant nutritional and metabolic health concerns worldwide, with major implications for long-term cardiovascular and metabolic morbidity.

The transition to university life is increasingly recognized as a vulnerable period for deterioration of dietary quality and unhealthy weight gain among young adults. This period is characterized by substantial lifestyle and behavioral changes, including increased dietary autonomy, irregular schedules, academic stress, financial limitations, and greater exposure to convenience-oriented food environments. Students commonly adopt less structured eating habits, including increased fast-food consumption, greater reliance on ready-to-eat meals, more frequent snacking, meal skipping, and reduced intake of fresh foods. Together with reduced physical activity, prolonged sedentary time, and sleep disruption, these behaviors may contribute to positive energy balance and progressive BMI increase during emerging adulthood [5,6,7,8].

Contemporary evidence indicates that university students are among the demographic groups with the highest exposure to fast foods, ultra-processed products, sugar-rich foods, and irregular meal patterns [9,10]. Food choices are frequently shaped by convenience, affordability, limited preparation time, campus food availability, and increasing reliance on food-delivery applications, while urban living environments further facilitate access to inexpensive energy-dense foods [11,12,13,14,15,16,17,18,19,20].

Parallel to these environmental changes, nutritional epidemiology has progressively shifted from isolated nutrient analysis toward broader dietary-pattern and behavioral-phenotype approaches. Increasing evidence suggests that overweight and obesity in young adults are influenced less by single dietary exposures and more by the clustering of multiple unhealthy eating behaviors, including frequent fast-food consumption, elevated sweets intake, inadequate water intake, low intake of fresh foods, and replacement of structured meals with snack-type or dessert-type products [21,22].

Importantly, these dietary behaviors rarely occur independently. Dietary-pattern studies consistently demonstrate that unhealthy eating practices aggregate into reproducible behavioral phenotypes associated with increased adiposity and cardiometabolic risk. The accumulation of several moderate-risk dietary behaviors may therefore exert stronger associations with overweight and obesity than isolated dietary variables analyzed separately [23,24]. This conceptual framework has stimulated growing interest in the use of composite dietary indicators and dietary-risk scores capable of capturing cumulative exposure to obesogenic eating patterns. Behavioral composite indices may improve risk stratification in younger populations by integrating multiple coexisting lifestyle exposures into simplified epidemiological tools [25,26].

Growing consumption of ultra-processed and convenience-oriented foods represents another important feature of contemporary dietary habits among young adults. These products are typically energy-dense, highly palatable, and rich in sugar, sodium, and saturated fat, characteristics that favor excess energy intake. High consumption of ultra-processed foods has consistently been associated with poorer dietary quality, increased adiposity, and adverse metabolic outcomes [27,28].

In Central and Eastern Europe, including Romania, rapid urbanization and ongoing nutrition transition have promoted westernized dietary patterns characterized by greater consumption of fast foods, ultra-processed products, and commercially prepared meals [29]. Consistent with these trends, recent Romanian studies have linked unhealthy dietary patterns with adverse metabolic risk markers among university students, highlighting the importance of investigating dietary behavioral profiles in this population [30].

Despite increasing international interest in obesity prevention among young adults, comparatively limited evidence is available regarding the clustering of convenience-oriented dietary behaviors among Romanian university students and their independent contribution to overweight and obesity risk after adjustment for demographic factors. In particular, few studies from this region have simultaneously evaluated behaviors such as fast-food consumption, sweets intake, meal replacement with desserts, water intake habits, and fresh-food consumption within the same analytical framework. Moreover, limited data are available regarding cumulative behavioral burden assessed through dietary-risk scores or the identification of dietary behavioral phenotypes using cluster-based approaches.

Against this background, the present cross-sectional study aimed to evaluate convenience-oriented dietary behaviors among Romanian university students and their relationship with BMI status and overweight/obesity risk. Specifically, we examined the independent associations between individual dietary behaviors and overweight/obesity and explored whether these behaviors clustered into distinct dietary behavioral phenotypes. It was hypothesized that convenience-oriented dietary patterns would be associated with increased overweight and obesity risk in this population. Beyond evaluating individual dietary behaviors, the study also sought to characterize cumulative dietary behavioral burden using an exploratory Dietary Risk Score and to identify distinct dietary behavioral phenotypes through cluster analysis.

## 2. Materials and Methods

### 2.1. Study Design, Setting, and Participants

A cross-sectional observational study was conducted among undergraduate students attending public universities in western Romania during regular academic semesters and outside examination periods. The study population derived from a larger prospective cohort investigating behavioral and nutritional changes during the transition to university life. Anthropometric and dietary-behavioral data included in the present analysis were obtained from the follow-up assessment corresponding to the end of the first academic year.

Eligible participants were students aged 18–24 years who were actively enrolled in undergraduate study programs. Exclusion criteria included chronic conditions or pharmacological treatments known to influence body weight, pregnancy, lactation, prescribed therapeutic diets, and incomplete anthropometric or questionnaire data.

Data were collected using a structured self-administered online questionnaire distributed through institutional electronic communication channels and social media platforms. The questionnaire was developed by the research team specifically for the present study to assess convenience-oriented dietary behaviors among university students. Item selection was informed by the study objectives and contemporary nutritional epidemiology literature, focusing on dietary behaviors commonly associated with overweight and obesity in young adults. Before implementation, the questionnaire was reviewed by investigators with expertise in nutrition and public health to ensure clarity, face validity, and relevance to the target population. Although the questionnaire was not formally psychometrically validated before use, all items were predefined and administered uniformly to all participants. Before questionnaire completion, participants received information regarding the study objectives, voluntary participation, confidentiality measures, and anonymous data processing procedures.

A total of 921 students fulfilled the eligibility criteria and constituted the analytical sample.

### 2.2. Anthropometric Assessment and BMI Classification

Anthropometric data were obtained through self-reported body weight and height collected using the online questionnaire. BMI was calculated as weight (kg) divided by height squared (m^2^). Participants were classified according to World Health Organization criteria as underweight (BMI < 18.5 kg/m^2^), normal weight (18.5–24.9 kg/m^2^), overweight (25.0–29.9 kg/m^2^), or obese (≥30.0 kg/m^2^)

For binary outcome analyses, overweight and obese categories were combined into a single “overweight/obese” category because of the relatively limited number of obese participants. A continuous BMI deviation index (Diff_BMI), defined as the difference between measured BMI and the midpoint of the normal-weight interval (21.7 kg/m^2^), was additionally calculated and used in sensitivity analyses.

### 2.3. Dietary and Behavioral Variables

Dietary behaviors were assessed using a structured self-administered questionnaire previously applied in Romanian young adults and behavioral nutrition research. The English version of the questionnaire is provided as Supplementary File S1.

The questionnaire evaluated weekly fast-food consumption (never; rarely; 1–2 times/week; ≥3 times/week), candy or chocolate intake (less than 2 times/week; 2 times/week; 3–4 times/week; daily), and fruit and vegetable consumption using identical response categories. Daily water intake was categorized as <1 L/day, 1–2 L/day, or >2 L/day.

Additional items assessed preference for the habit of replacing meals with desserts (never; sometimes; frequently). Meal replacement with desserts was defined as the habitual substitution of a regular main meal with sweet foods or desserts rather than the occasional consumption of desserts following a meal. For the regression analyses, responses were subsequently dichotomized as frequent versus never/sometimes.

### 2.4. Construction of the Dietary Risk Score

A composite Dietary Risk Score (DRS) was constructed a priori using five dietary behaviors previously associated with obesogenic eating patterns in young adults. The DRS was developed a priori as an exploratory composite indicator intended to capture the cumulative burden of convenience-oriented dietary behaviors rather than to quantify the contribution of individual dietary exposures. The five selected components were chosen based on previous evidence linking these behaviors with unhealthy dietary patterns and excess body weight in young adults. Equal weighting was applied because there is currently no established evidence supporting differential weighting of these behaviors within a composite score, while equal weighting improves transparency, simplicity, and reproducibility. The selected components were chosen a priori based on existing evidence linking these behaviors to unhealthy dietary patterns and excess body weight in young adults. Equal weighting was applied to maximize simplicity, transparency, and ease of interpretation. One point was assigned for each of the following conditions: (a) fast-food consumption ≥3 times/week; (b) low fruit and vegetable intake (≤2 times/week); (c) daily water intake <1 L; (d) daily candy/chocolate consumption; and (e) frequent replacement of meals with desserts. The threshold for fruit and vegetable consumption was selected to identify participants reporting the lowest frequency of intake within the study population based on the available questionnaire response categories and was not intended to represent adherence to, or deviation from, current dietary guideline recommendations. Similarly, the remaining thresholds were based on the predefined response categories of the study questionnaire and were established a priori to reflect meaningful behavioral categories rather than data-driven cut-offs.

The DRS ranged from 0 to 5, with higher scores indicating greater accumulation of convenience-oriented dietary risk behaviors. For stratified analyses, DRS values ≥3 were collapsed into a single “3+” category to preserve statistical power.

### 2.5. Statistical Analysis

Continuous variables are presented as mean ± standard deviation (SD) and median [interquartile range (IQR)], whereas categorical variables are reported as absolute frequencies and percentages. Group comparisons of categorical variables across sex, residence environment, and BMI categories were performed using Pearson’s chi-square test, with effect size quantified using Cramer’s V.

Associations among dietary variables were evaluated using Spearman rank-order correlation coefficients. Correlation matrices were additionally visualized using heatmap representations.

Crude odds ratios (ORs) with 95% confidence intervals (CIs) were calculated for dietary behaviors in relation to overweight/obesity. Multivariable binary logistic regression models were constructed using overweight/obesity as the dependent outcome and dietary behaviors as predictors, with adjustment for sex, age group (18–20 vs. 21–24 years), and residence environment (urban vs. rural). A complementary model evaluating predictors of normal-weight status was also developed.

Exploratory sex-stratified analyses were additionally performed to evaluate the consistency of the observed associations across women and men. Formal multiplicative interaction terms were included in the multivariable models to assess potential effect modification by sex.

For DRS analyses, both continuous (per one-point increase) and categorical approaches (reference: DRS = 0) were evaluated. Model performance was assessed using the Hosmer–Lemeshow goodness-of-fit test, Nagelkerke’s R^2^, and the area under the receiver operating characteristic curve (AUC).

Dietary behavioral phenotypes were identified using k-means cluster analysis based on the five core dietary variables included in the DRS. The optimal number of clusters was selected using elbow-method optimization. Hierarchical Ward clustering was additionally performed as a complementary exploratory clustering approach to examine the consistency of the identified cluster structure.

Sensitivity analyses included exclusion of underweight participants, obesity-only outcome models, ordinal logistic regression analyses using BMI categories, and multiple linear regression analyses using continuous Diff_BMI as outcome.

A two-sided *p* < 0.05 was considered statistically significant. All statistical analyses were conducted in R (v. 4.3.2; R Foundation for Statistical Computing, Vienna, Austria) and JASP (v. 0.18.3; JASP Team, University of Amsterdam, Amsterdam, The Netherlands).

### 2.6. Ethical Considerations

Participation in the study was entirely voluntary. Prior to enrolment, all participants were informed about the study aims, data collection procedures, confidentiality safeguards, and the intended scientific use of the collected information. Electronic written informed consent was obtained from each participant before access to the questionnaire was granted.

The study protocol received approval from the Ethics Committee of the “Victor Babeș” University of Medicine and Pharmacy Timișoara (approval no. 95/4 October 2021). All study procedures were conducted in accordance with the Declaration of Helsinki and established principles of good biomedical research practice.

## 3. Results

### 3.1. Participant Characteristics According to BMI Category

The analytical sample included 921 university students, of whom 558 (60.6%) were women and 363 (39.4%) were men. According to the WHO BMI classification, 59 participants (6.4%) were underweight, 640 (69.5%) had normal weight, and 222 (24.1%) were classified as overweight/obesity.

The prevalence of overweight/obesity according to participant characteristics is presented in Table 1. Overweight/obesity was more prevalent among men than women (32.5% vs. 18.6%; *p* < 0.001; V = 0.191), among students aged 21–24 years than those aged 18–20 years (30.3% vs. 17.4%; *p* < 0.001; V = 0.154), and among students living in urban compared with rural areas (28.4% vs. 15.2%; *p* < 0.001; V = 0.150).

Among the dietary behaviors examined, the largest differences in overweight/obesity prevalence were observed for frequent meal replacement with desserts (64.0% vs. 19.2%; *p* < 0.001; V = 0.239) and frequent fast-food consumption (38.0% vs. 21.2%; *p* < 0.001; V = 0.148).

The Dietary Risk Score (DRS) also differed significantly according to BMI status (Table 1; Mann–Whitney U test, *p* = 0.0017), with participants with overweight/obesity exhibiting higher mean DRS values than the overall study population.

### 3.2. Distribution of Convenience-Oriented Dietary Behaviors

The distribution of the analyzed dietary behaviors is presented in Supplementary Table S1. Frequent fast-food consumption (≥3 times/week) was reported by 17.2% of participants, daily candy or chocolate intake by 26.2%, frequent meal replacement with desserts by 10.9%, and suboptimal daily water intake (<1 L/day) by 22.8% of students.

Several dietary behaviors differed significantly according to sex and residence environment (Supplementary Table S1). Fast-food frequency and daily water intake differed by sex (both *p* < 0.05), whereas sweets intake and meal replacement with desserts differed according to residence environment (both *p* < 0.05). The largest differences according to overweight/obesity status were observed for frequent meal replacement with desserts (V = 0.340) and fast-food consumption (V = 0.200).

**Figure 1:**
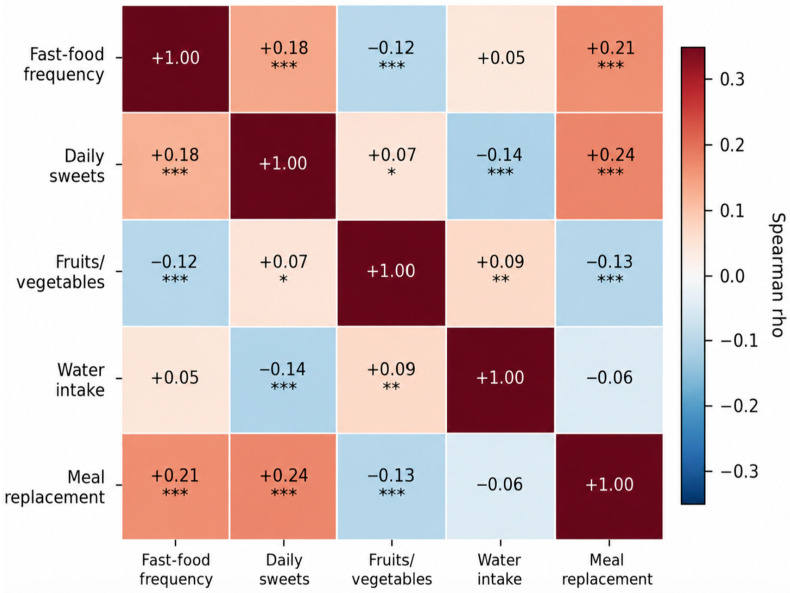
Spearman correlation heatmap of the six core dietary behaviors included in the behavioral-pattern analysis (N = 921). Positive correlations indicate co-occurrence of fast-food consumption, sweets intake, and meal replacement with desserts, whereas fruit and vegetable intake showed inverse correlations with these behaviors. Statistical significance levels displayed within the heatmap are coded as follows: ***, *p* < 0.001; **, *p* < 0.01; and * *p* < 0.05. FF, fast-food frequency; SWEETS, daily sweets intake; FV, fruit and vegetable intake; WATER, daily water intake; MR, meal replacement with desserts.

Correlations among the five core dietary behaviors are illustrated in Figure 1, with the corresponding Spearman correlation coefficients provided in Supplementary Table S2. Overall, correlations were weak to moderate, indicating that the analyzed dietary behaviors were related but represented complementary rather than redundant dimensions of convenience-oriented eating patterns. This supported their subsequent inclusion in the exploratory Dietary Risk Score and cluster analysis. The strongest positive correlations were observed between meal replacement with desserts and daily sweets intake (rho = 0.242; *p* < 0.001), and between meal replacement with desserts and fast-food consumption (rho = 0.206; *p* < 0.001), whereas fruit and vegetable intake showed weak inverse correlations with these behaviors. Daily water intake demonstrated comparatively weak correlations with the remaining dietary variables.

### 3.3. BMI Distribution Across Convenience-Oriented Dietary Behaviors

The distribution of BMI categories according to the principal dietary behaviors is presented in Table 2. Significant associations with BMI status were observed for fast-food frequency (*p* < 0.001; V = 0.148), daily candy/chocolate intake (*p* = 0.024; V = 0.089), frequent meal replacement with desserts (*p* < 0.001; V = 0.239), and the Dietary Risk Score (DRS) (*p* < 0.001; V = 0.147), whereas fruit and vegetable intake (*p* = 0.808) and daily water intake (*p* = 0.072) were not significantly associated with BMI category.

The largest differences in overweight/obesity prevalence were observed for frequent meal replacement with desserts (64.0% vs. 14.5% among students who never replaced meals with desserts) and frequent fast-food consumption (38.0% vs. 14.9% among students reporting rare consumption). Linear-by-linear association analyses supported significant ordinal trends for fast-food frequency (*p*-trend < 0.001), meal replacement with desserts (*p*-trend < 0.001), daily candy/chocolate intake (*p*-trend = 0.019), and DRS levels (*p*-trend < 0.001), whereas no significant trends were observed for fruit and vegetable intake or daily water intake (Table 2).

### 3.4. Stratified Analyses According to Sex and Age

Exploratory sex-stratified analyses are presented in Table 3. The direction and magnitude of the associations were generally consistent across women and men, and formal interaction testing did not indicate significant effect modification by sex (all interaction *p* > 0.05). Frequent meal replacement with desserts showed the strongest association with overweight/obesity in both sexes, whereas frequent fast-food consumption was significantly associated with overweight/obesity only among women, although the direction of the association was similar in men.

The prevalence of overweight/obesity was higher among students aged 21–24 years than among those aged 18–20 years (30.3% vs. 17.4%; *p* < 0.001; V = 0.154). Frequent meal replacement with desserts was also more prevalent among older students (*p* = 0.021), whereas fast-food frequency, DRS values, and the age × DRS interaction term did not differ significantly between age groups (all *p* > 0.05).

### 3.5. Individual Behavioral Components Associated with Overweight and Obesity

Multivariable logistic regression analyses evaluating the associations between individual dietary behaviors and overweight/obesity are presented in Table 4. A dose-dependent association was observed for fast-food consumption. Compared with students reporting rare fast-food consumption, those consuming fast-food 1–2 times/week had higher adjusted odds of overweight/obesity (aOR 2.19; 95% CI 1.50–3.21; *p* < 0.001), while consumption ≥3 times/week was associated with nearly fourfold higher odds (aOR 3.68; 95% CI 2.33–5.79; *p* < 0.001). The corresponding ordinal trend was also statistically significant (OR per category increase 1.76; 95% CI 1.44–2.17; *p* < 0.001).

Daily candy or chocolate intake was not independently associated with overweight/obesity (aOR per category increase 1.08; 95% CI 0.92–1.28; *p* = 0.352). In contrast, frequent meal replacement with desserts demonstrated the strongest association with overweight/obesity, with more than tenfold higher adjusted odds compared with students who never replaced meals with desserts (aOR 10.55; 95% CI 6.40–17.39; *p* < 0.001). A significant dose-dependent association was also observed across meal replacement categories (aOR per exposure level 2.96; 95% CI 2.30–3.81; *p* < 0.001).

Ordinal logistic regression analyses using the four-level BMI classification as the outcome yielded consistent findings, demonstrating a significant association between increasing meal replacement frequency and progressively higher BMI categories (aOR 2.08; 95% CI 1.64–2.63; *p* < 0.001).

### 3.6. Protective Dietary Factors Associated with Normal Weight

A multivariable logistic regression model evaluating factors associated with normal-weight status is presented in Table 5. Underweight participants were excluded from this analysis, resulting in an analytical sample of 862 students.

Daily fruit and vegetable intake (aOR 0.83; 95% CI 0.59–1.16; *p* = 0.269) and adequate water intake (≥2 L/day) (aOR 0.81; 95% CI 0.54–1.22; *p* = 0.319) were not independently associated with normal-weight status.

Among the demographic covariates, female sex (aOR 1.77; 95% CI 1.28–2.46; *p* < 0.001), younger age (18–20 years) (aOR 2.25; 95% CI 1.62–3.14; *p* < 0.001), and rural residence (aOR 2.41; 95% CI 1.64–3.53; *p* < 0.001) were independently associated with normal-weight status.

### 3.7. Identification of Dietary Behavioral Phenotypes

**Figure 2:**
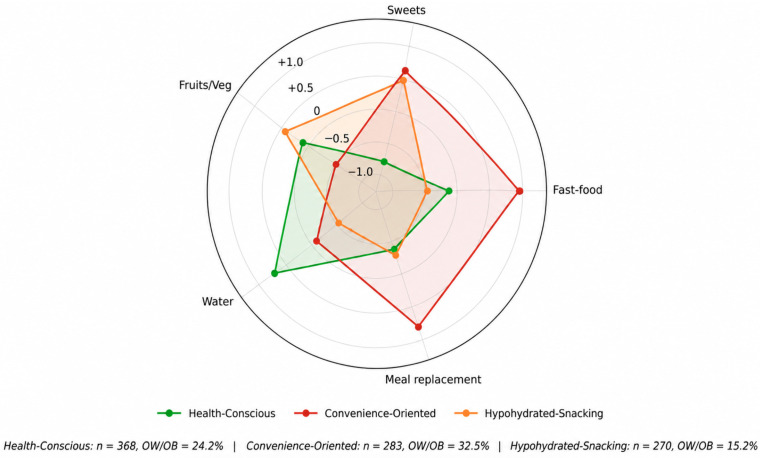
Radar plot of the three dietary behavioral phenotypes identified by k-means cluster analysis. Standardized z-scores of the five core dietary behaviors are shown for the Convenience-Oriented, Health-Conscious, and Low-Water-Intake-Snacking clusters. The Convenience-Oriented cluster demonstrated the highest prevalence of overweight/obesity and the highest Dietary Risk Score. FF, fast-food frequency; SWEETS, daily sweets intake; FV, fruit and vegetable intake; WATER, daily water intake; MR, meal replacement with desserts; DRS, Dietary Risk Score.

K-means cluster analysis based on the five core dietary behaviors included in the Dietary Risk Score (DRS) identified three distinct dietary behavioral phenotypes. The three-cluster solution was selected using elbow-method optimization, and hierarchical Ward clustering yielded a similar cluster structure. The characteristics of the identified clusters are summarized in Table 6 and illustrated in Figure 2.

The Convenience-Oriented phenotype included 283 students (30.7%) and was characterized by higher fast-food consumption, sweets intake, and meal replacement with desserts, together with lower fruit and vegetable intake. This cluster exhibited the highest mean DRS (1.98 ± 1.00) and the highest prevalence of overweight/obesity (32.5%).

The Health-Conscious phenotype comprised 368 students (40.0%) and was characterized by higher water intake and lower sweets intake and meal replacement with desserts. Participants in this cluster had the lowest mean DRS (0.35 ± 0.53), while the prevalence of overweight/obesity was 24.2%.

The Low-Water Intake Snacking phenotype included 270 students (29.3%) and was characterized by lower water intake, low fast-food consumption, moderate sweets intake, and relatively higher fruit and vegetable intake. This cluster demonstrated the lowest prevalence of overweight/obesity (15.2%).

Differences among clusters were statistically significant for BMI distribution (*p* < 0.001), DRS distribution (*p* < 0.001), and sex distribution (*p* = 0.027).

### 3.8. Dietary Risk Score and Dose–Response Relationship

**Figure 3:**
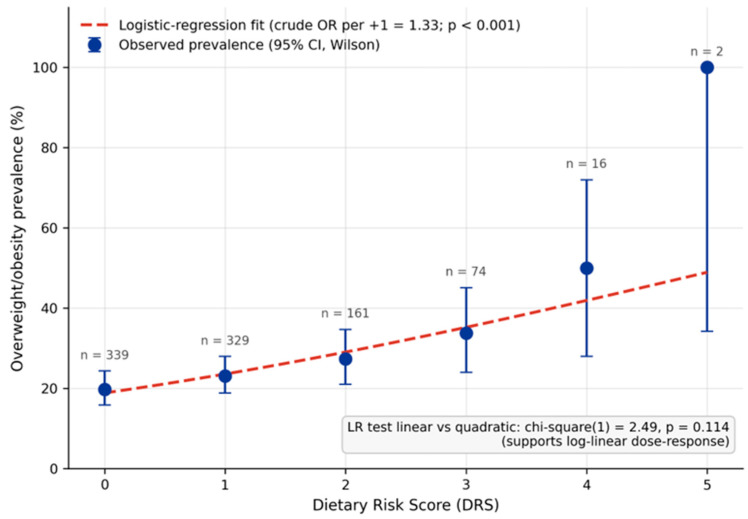
Dose–response relationship between Dietary Risk Score (DRS) and overweight/obesity prevalence. Points represent observed overweight/obesity prevalence across DRS categories with Wilson 95% confidence intervals, while the dashed line indicates logistic-regression-predicted probabilities. Overweight/obesity prevalence increased progressively from 19.8% at DRS = 0 to 50.0% at DRS = 4. CI, confidence interval; DRS, Dietary Risk Score.

The distribution of the Dietary Risk Score (DRS) and its association with overweight/obesity are presented in Figure 3. Overall, 339 students (36.8%) had a DRS of 0, 329 (35.7%) a DRS of 1, 161 (17.5%) a DRS of 2, 74 (8.0%) a DRS of 3, 16 (1.7%) a DRS of 4, and 2 participants (0.2%) reached the maximum score of 5.

The observed prevalence of overweight/obesity increased across DRS categories, ranging from 19.8% among students with DRS = 0 to 50.0% among those with DRS = 4 (Figure 3). Both the overall association (*p* < 0.001; V = 0.147) and the linear-by-linear trend analysis (*p*-trend < 0.001) were statistically significant.

In logistic regression analyses, each one-point increase in DRS was associated with higher odds of overweight/obesity in both crude (OR 1.33; 95% CI 1.15–1.53; *p* < 0.001) and adjusted models (aOR 1.43; 95% CI 1.23–1.66; *p* < 0.001). Compared with students with DRS = 0, those with DRS = 4 had approximately fourfold higher adjusted odds of overweight/obesity (aOR 4.06; 95% CI 1.43–11.50).

The likelihood-ratio test comparing linear and quadratic DRS specifications was not statistically significant (*p* = 0.114), indicating no evidence of departure from linearity across the observed DRS range. The DRS demonstrated modest discrimination for overweight/obesity (AUC 0.572; 95% CI 0.550–0.633).

### 3.9. Final Multivariable Model for Overweight and Obesity

**Figure 4:**
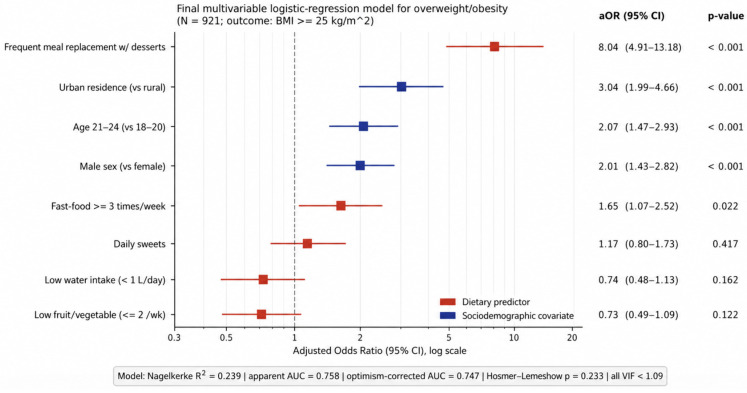
Forest plot of adjusted odds ratios (aORs) and 95% confidence intervals derived from the final multivariable logistic regression model for overweight/obesity (N = 921; outcome: BMI ≥ 25 kg/m^2^). Red markers indicate dietary predictors, and blue markers indicate sociodemographic covariates. Frequent meal replacement with desserts showed the strongest independent association with overweight/obesity after adjustment for all dietary and demographic variables. aOR, adjusted odds ratio; AUC, area under the receiver operating characteristic curve; BMI, body mass index; CI, confidence interval; VIF, variance inflation factor.

The final multivariable logistic regression model for overweight/obesity is presented in Table 7 and Figure 4. After simultaneous adjustment for all dietary variables and demographic covariates, frequent meal replacement with desserts and fast-food consumption ≥3 times/week remained independently associated with overweight/obesity.

Frequent meal replacement with desserts showed the strongest association with overweight/obesity (aOR 8.04; 95% CI 4.91–13.18; *p* < 0.001), whereas fast-food consumption ≥3 times/week was associated with approximately 1.6-fold higher odds of overweight/obesity (aOR 1.65; 95% CI 1.07–2.52; *p* = 0.022). Daily sweets intake, low fruit and vegetable consumption, and low water intake were not independently associated with overweight/obesity.

Male sex (aOR 2.01; 95% CI 1.43–2.82), age 21–24 years (aOR 2.07; 95% CI 1.47–2.93), and urban residence (aOR 3.04; 95% CI 1.99–4.66) were also independently associated with overweight/obesity.

The model demonstrated adequate calibration (Hosmer–Lemeshow *p* = 0.233) and acceptable discrimination (AUC = 0.758). Bootstrap internal validation yielded an optimism-corrected AUC of 0.747, while all variance inflation factors were below 1.09 (Supplementary Table S3), indicating no evidence of problematic multicollinearity.

### 3.10. Sensitivity Analyses

The complete regression outputs for the sensitivity analyses are provided in Supplementary Table S4, while a summary of the principal findings is presented in Supplementary Table S5. Excluding underweight participants (N = 862) yielded findings consistent with the primary analysis, with frequent meal replacement with desserts (aOR 8.07; 95% CI 4.88–13.35; *p* < 0.001) and fast-food consumption ≥3 times/week (aOR 1.64; 95% CI 1.07–2.52; *p* = 0.024) remaining independently associated with overweight/obesity.

Analyses restricted to obesity (BMI ≥ 30 kg/m^2^), alternative DRS categorization, multiple linear regression using continuous Diff_BMI, and ordinal logistic regression produced generally consistent results, supporting the robustness of the principal findings. Across all sensitivity analyses, frequent meal replacement with desserts remained the most consistent dietary correlate of overweight/obesity, whereas fast-food consumption also showed consistent positive associations.

## 4. Discussion

The present study identified a consistent association between convenience-oriented dietary behavioral patterns and overweight/obesity risk among Romanian university students during the transition to independent adult life. Rather than reflecting a single isolated dietary exposure, excess weight was associated with the accumulation and clustering of multiple convenience-related behaviors, particularly frequent fast-food consumption, and replacement of structured meals with desserts. The integrated analytical framework employed in the present study, including dietary-pattern clustering, composite risk-score modeling, multivariable regression, and sensitivity analyses, supports the concept that overweight/obesity is more strongly associated with broader behavioral phenotypes than with isolated nutritional variables alone.

One of the most important findings was the identification of a distinct convenience-oriented dietary phenotype characterized by high fast-food intake, elevated sweets consumption, frequent replacement of meals with desserts, and lower fruit and vegetable intake. This phenotype demonstrated both the highest Dietary Risk Score (DRS) values and the highest prevalence of overweight/obesity. These findings align with contemporary nutritional epidemiology models indicating that unhealthy dietary behaviors tend to cluster into reproducible lifestyle phenotypes associated with increased adiposity and metabolic risk [31]. Such behavioral aggregation is particularly relevant during university transition, a developmental period frequently characterized by reduced parental supervision, constrained budgets, irregular schedules, academic stress, and increased exposure to hyper-palatable convenience foods [9]. Although the identified dietary behavioral phenotypes were supported by two complementary clustering approaches, they should be considered exploratory and warrant replication in independent populations.

The clinical relevance of behavioral clustering is important because nutritional interventions frequently focus on isolated dietary targets without accounting for the synergistic accumulation between multiple moderate-risk behaviors. In the present study, cumulative exposure to several convenience-oriented behaviors appeared to amplify overweight/obesity risk beyond the effect of individual dietary exposures alone. Similar clustering dynamics have been reported in studies examining dietary patterns and cardiometabolic risk among young adults, where aggregated behavioral profiles demonstrated stronger associations with BMI than isolated food-frequency variables [32,33,34].

The associations observed for meal replacement with desserts and frequent fast-food consumption may reflect broader lifestyle characteristics linked to convenience-oriented eating patterns. University students frequently prioritize affordability, rapid preparation, portability, and satiety over nutritional quality, particularly during periods of academic stress, financial limitation, and time scarcity. Such circumstances may facilitate greater dependence on ready-to-consume and industrially prepared meals requiring minimal cooking skills or meal planning. Recent evidence indicates that college students often rely on convenience meals and fast food when academic demands and financial insecurity limit access to healthier choices. This interpretation is additionally supported by experimental evidence demonstrating that ultra-processed diets may increase spontaneous energy intake and body weight under controlled conditions [35,36]. An additional explanation may involve reduced food literacy and limited cooking autonomy among students living independently for the first time. Previous studies have shown that lower cooking confidence and reduced meal-planning skills are associated with poorer dietary quality and increased dependence on ready-to-eat foods in younger populations [37].

Frequent replacement of meals with desserts represented another major behavioral correlate of overweight/obesity. This variable should be interpreted as a marker of disrupted meal organization and reliance on sweet foods in place of complete meals rather than simply a preference for desserts. However, because of the cross-sectional design, the direction of this association cannot be determined. Reverse causation is possible, and individuals with overweight or obesity may be more likely to adopt irregular eating patterns, including meal replacement behaviors, whether as a consequence of established dietary habits, weight-management attempts, or other lifestyle factors. Consequently, the present findings should be interpreted as demonstrating an association rather than a causal relationship. This finding is particularly relevant because replacing structured meals with sweets likely reflects broader disruption of satiety regulation and meal organization rather than isolated sugar consumption alone. Desserts and confectionery products are generally energy dense, rapidly consumed, highly palatable, and relatively poor in protein and fiber, characteristics that may contribute to incomplete satiety and compensatory caloric intake later during the day [38]. Irregular meal structure and meal-skipping behaviors have also been associated with altered metabolic regulation, poorer circadian eating organization, and increased obesity risk among young adults [39].

The present findings are consistent with previous evidence suggesting that irregular meal structure and timing are associated with adiposity risk beyond total caloric intake alone. Contemporary chrononutrition research indicates that irregular or mistimed food intake may disrupt circadian metabolic regulation and contribute to obesity-related metabolic risk [40].

University students frequently develop irregular eating schedules secondary to academic obligations, social activities, time scarcity, and financial constraints. Under such conditions, desserts and snack products may progressively replace nutritionally balanced meals because they are inexpensive, rapidly accessible, and emotionally rewarding. Recent evidence among university populations indicates that barriers such as cost, time pressure, food availability, limited cooking skills, and academic routines contribute to unhealthy eating behaviors and reduced dietary quality [12]. Such substitution behaviors may additionally interact with stress-related and reward-driven eating patterns. Emotional eating has been associated with increased intake of hyper-palatable energy-dense foods and with adverse nutritional-status indicators in younger populations [41].

Although psychological variables were not directly evaluated in the present study, emerging evidence suggests that academic stress and emotional burden frequently coexist with greater consumption of sweets, fast foods, and ultra-processed snack products among university students [42,43].

Fast-food consumption demonstrated a clear dose–response relationship with overweight/obesity risk in both crude and adjusted analyses. Students reporting fast-food intake at least three times weekly had approximately twofold higher adjusted odds of overweight/obesity, while intermediate-frequency consumers also demonstrated elevated risk. This graded association strengthens the biological plausibility of the observed relationship. Fast-food meals are typically characterized by high caloric density, large portion sizes, elevated saturated-fat and sodium content, and relatively low micronutrient density. In addition, fast-food environments frequently encourage passive overconsumption through hyper-palatability, aggressive marketing, and convenience-based accessibility [44].

Importantly, the observed relationship persisted after adjustment for multiple coexisting dietary behaviors, suggesting that fast-food intake may exert an independent contribution to adiposity risk within broader unhealthy dietary patterns. This interpretation is consistent with longitudinal evidence demonstrating that greater fast-food consumption during adolescence predicts increased weight gain during the transition to adulthood [45]. Another possible explanatory mechanism involves the reward-related properties of commercially prepared fast foods and desserts. Ultra-processed products are often engineered to maximize sensory appeal through combinations of sugar, fat, sodium, flavor enhancers, and texture modifiers that may reinforce repetitive intake behaviors and hedonic eating patterns extending beyond physiological hunger regulation [46]. Experimental metabolic studies further demonstrate that ultra-processed diets may increase spontaneous caloric intake and induce weight gain under controlled feeding conditions [36].

The DRS provided additional insight into the cumulative nature of dietary risk. The observed association between the DRS and overweight/obesity should not be interpreted as implying that every individual component contributed equally or independently to risk. Rather, the DRS was intentionally developed a priori as an exploratory composite indicator of cumulative dietary behavioral burden, designed to capture the co-occurrence of multiple convenience-oriented behaviors. The weak-to-moderate correlations observed among the individual dietary behaviors further support this approach, indicating that the selected variables captured related but complementary aspects of convenience-oriented eating patterns rather than measuring the same underlying construct. Equal weighting was applied to maximize simplicity, transparency, and reproducibility in the absence of evidence supporting differential weighting of the individual components. Composite indices can remain informative even when some individual components are not independently associated with the outcome because they capture the cumulative burden and co-occurrence of multiple dietary behaviors rather than the isolated effect of each component. This conceptual approach is consistent with contemporary nutritional epidemiology, which emphasizes the assessment of overall dietary patterns and cumulative dietary behaviors rather than isolated dietary exposures. Accordingly, the principal contribution of the present study does not lie in confirming previously reported associations between individual dietary behaviors and overweight/obesity, but in demonstrating that convenience-oriented dietary behaviors can be integrated into an exploratory composite score reflecting cumulative behavioral burden. Together with the complementary cluster analysis, this approach provides a broader characterization of dietary behavioral patterns during the transition to university than analyses of individual dietary behaviors alone. In the present study, the strongest contributors to the observed association were frequent meal replacement with desserts and fast-food consumption, while the remaining components may have improved the characterization of overall dietary behavior despite weaker individual associations.

Overweight/obesity prevalence increased progressively across DRS categories, while the DRS remained significantly associated with overweight/obesity across sensitivity analyses. This dose–response gradient supports the ability of the DRS to capture cumulative dietary behavioral burden within the present study population and suggests that cumulative behavioral exposures may be more informative than isolated dietary behaviors. Nevertheless, the DRS should be interpreted as an exploratory research tool rather than a validated clinical instrument. Although the observed dose–response relationship supports its potential utility for capturing cumulative dietary behavioral burden, external validation in independent populations is necessary before broader application can be recommended. Contemporary evidence indicates that clustering of multiple moderate-risk lifestyle behaviors may exert stronger associations with overweight and obesity than single behaviors analyzed independently [47].

These findings are also consistent with multifactorial obesity frameworks emphasizing that excess weight gain emerges from the accumulation of numerous behavioral and environmental determinants rather than from a single dominant dietary factor [48]. In this context, increasing attention has been directed toward early identification of obesogenic behavioral profiles and toward preventive strategies targeting multiple coexisting lifestyle risk factors simultaneously, particularly in younger populations in whom long-term dietary trajectories are still being established [49].

Interestingly, lower-frequency fruit and vegetable consumption and inadequate water intake were not significantly associated with BMI category individually and did not retain significance in the multivariable analyses. This finding should not be interpreted as evidence that these behaviors lack metabolic relevance. It should also be considered that fruit and vegetable consumption was assessed using frequency-based questionnaire categories rather than quantitative measures of daily intake or adherence to dietary recommendations, which may have limited the ability to detect independent associations. Rather, it likely reflects the dominant influence of broader convenience-oriented dietary patterns that statistically absorbed weaker individual associations when simultaneously modeled. Contemporary dietary-pattern research increasingly suggests that obesity-related risk is more strongly determined by the co-occurrence between coexisting eating behaviors than by isolated dietary variables analyzed independently [50]. Similar attenuation effects have been described in multivariable dietary-pattern analyses where associations for isolated protective behaviors weaken after adjustment for broader ultra-processed-food exposure and meal-pattern characteristics exerting stronger obesogenic influence [19].

The absence of significant independent associations for fruit and vegetable intake may additionally reflect the relatively low variability of this behavior within the cohort and the modest effect sizes generally reported in nutritional epidemiology studies evaluating protective dietary exposures [51]. Furthermore, protective dietary factors may exert weaker short-term effects on BMI than highly energy-dense convenience-food exposures, particularly in younger individuals with relatively preserved metabolic flexibility [52]. Another possible explanation is that BMI incompletely captures the broader metabolic consequences of water intake and fruit/vegetable intake. Considerable metabolic heterogeneity exists within BMI categories, and normal-weight individuals may still present unfavorable dietary patterns or early metabolic alterations that are not fully reflected by anthropometric classification alone [53].

Consequently, the absence of independent BMI associations should not be interpreted as absence of clinical relevance for these dietary behaviors [54]. Emerging evidence further suggests that nutritional interventions may influence body composition and metabolic health independently of major BMI changes alone. Improvements in dietary quality and targeted nutritional supplementation have been associated with favorable body-composition modifications and better health-related outcomes, supporting the concept that BMI may incompletely reflect the broader physiological impact of dietary behaviors [30].

Sex-, age-, and residence-related findings also deserve attention. Male students demonstrated significantly higher odds of overweight/obesity, consistent with evidence indicating that young men frequently exhibit lower nutritional awareness, poorer dietary quality, and reduced engagement in weight-management behaviors compared with women [55,56]. Additional studies among young adults have similarly reported that women more frequently engage in dietary monitoring and weight-control practices, whereas men often display higher consumption of energy-dense foods and lower adherence to healthy dietary recommendations [57].

Older students (21–24 years) also demonstrated increased overweight/obesity prevalence. This finding may reflect cumulative exposure to convenience-oriented dietary environments and progressive normalization of obesogenic eating patterns across university years. Emerging adulthood is increasingly recognized as a critical developmental period during which lifestyle behaviors become progressively consolidated, with potential long-term implications for future health trajectories [58]. Recent longitudinal evidence further suggests that dietary patterns established during emerging adulthood may persist across later life stages and remain associated with future weight status [59].

Urban residence independently predicted overweight/obesity in the adjusted model. Urban food environments generally provide greater availability of fast-food outlets, food-delivery services, and ready-to-consume products while simultaneously encouraging more sedentary lifestyles. Contemporary evidence suggests that environmental determinants strongly shape dietary behaviors among students, particularly within highly urbanized settings characterized by abundant convenience-food availability [60,61]. Recent European evidence further indicates that urbanization is associated with increased exposure to obesogenic food environments and greater accessibility of highly processed convenience foods among younger populations [4].

The present findings should additionally be interpreted within the broader framework of the ongoing nutrition transition observed across many European countries, including Romania. Contemporary food environments are increasingly characterized by widespread availability of ultra-processed foods, digital food-access systems, and progressive displacement of traditional dietary practices [20]. Increasing urbanization, widespread food-delivery platforms, prolonged screen exposure, and greater accessibility of ready-to-consume products may further reinforce convenience-oriented food choices among younger populations [62].

The present findings carry several practical implications for university health promotion. Nutritional interventions targeting students should move beyond generalized advice to “eat healthier” and instead address the behavioral mechanisms underlying convenience-oriented eating patterns. Screening approaches focused on frequent fast-food intake, meal replacement with desserts, convenience-oriented eating behaviors may provide efficient strategies for identifying students at elevated obesity risk. Universities could additionally improve access to affordable fresh meals, implement structured nutritional education programs, and promote practical competencies such as food preparation and meal planning.

This study has several important strengths. The sample size was relatively large, multiple dietary behaviors were simultaneously evaluated, and the analytical framework integrated dietary-pattern clustering, composite-score modeling, multivariable regression, and sensitivity analyses. The consistency of the principal findings across alternative statistical approaches further strengthens confidence in the robustness of the observed associations. The study additionally contributes novel epidemiological data from Romania, a region where university dietary-transition research remains comparatively limited.

It is also noteworthy that the principal associations remained directionally stable across multiple sensitivity analyses, including exclusion of underweight participants, obesity-only models, ordinal logistic regression, and analyses using continuous BMI deviation. Such consistency reduces the likelihood that the findings merely reflect statistical artifacts related to BMI categorization and strengthens the internal validity of the observed relationships. Replication of directionally consistent associations across alternative analytical strategies is increasingly regarded as an important indicator of robustness and reproducibility in nutritional epidemiology [63].

Several limitations should nevertheless be acknowledged. The cross-sectional de-sign precludes conclusions regarding causality or temporal directionality. Although the multivariable models were adjusted for sex, age, and residence environment, residual confounding cannot be excluded. Information on other factors known to influence body weight, including physical activity, sedentary behavior, sleep duration, alcohol consumption, socioeconomic status, and psychological stress, was not available and therefore could not be included in the analyses. Consequently, part of the observed associations may be explained by these unmeasured variables. Future longitudinal studies incorporating a broader range of lifestyle and socioeconomic factors are warranted. Because dietary behaviors and BMI were assessed simultaneously, it cannot be determined whether convenience-oriented dietary behaviors contributed to overweight/obesity or whether existing weight status influenced dietary choices. Reverse causality therefore cannot be excluded Both anthropometric and dietary variables were self-reported and therefore subject to recall and reporting biases. In particular, self-reported body weight and height may have resulted in some degree of BMI misclassification due to underreporting of weight or overreporting of height. In addition, the dietary questionnaire was developed specifically for this study and, although reviewed for face validity by the research team before implementation, it did not undergo formal psychometric validation. Consequently, some degree of measurement error or misclassification cannot be excluded. The questionnaire evaluated frequency-based behaviors rather than detailed caloric intake, portion size, physical activity, sleep characteristics, alcohol consumption, socioeconomic indicators, stress-related factors, or other psychological variables. Furthermore, fruit and vegetable consumption was assessed using frequency-based categories rather than quantitative measures of servings or portions, limiting direct comparison with current dietary guideline recommendations. Thus, residual confounding cannot be excluded and some of the observed associations may be partly influenced by unmeasured lifestyle and environmental determinants of body weight. In addition, the Dietary Risk Score was developed specifically for this study as an exploratory composite measure using equally weighted behavioral components and questionnaire-based cut-off values. Although this approach improved transparency and ease of interpretation, alternative weighting strategies or threshold definitions may produce different results. Consequently, the DRS should be regarded as an exploratory research tool that requires external validation in independent populations before broader application. The highest DRS categories contained relatively few participants, resulting in less precise estimates and wider confidence intervals. Therefore, findings for these categories should be interpreted cautiously until confirmed in larger studies. Accordingly, its applicability beyond the present population should be interpreted with caution until further validation studies are conducted. Finally, participants were recruited exclusively from public universities in western Romania, and extrapolation to other populations should therefore be performed cautiously. Nevertheless, the consistency of the principal findings across multiple analytical approaches and sensitivity analyses supports the robustness of the observed associations.

Future longitudinal studies should evaluate whether convenience-oriented dietary phenotypes predict subsequent BMI trajectories and cardiometabolic outcomes during emerging adulthood. More detailed assessment of food-processing level, emotional eating, sleep, stress, physical activity, alcohol consumption, and socioeconomic factors would further clarify the mechanisms linking convenience-oriented behaviors with obesity risk and help reduce residual confounding in future investigations. Intervention studies specifically targeting meal replacement behaviors, fast-food exposure, and reliance on ultra-processed convenience meals may provide valuable strategies for obesity prevention in university settings.

## 5. Conclusions

In this cohort of Romanian university students aged 18–24 years, convenience-oriented dietary behaviors were associated with overweight and obesity status. Frequent replacement of meals with desserts and regular fast-food consumption emerged as the dietary behaviors most consistently associated with overweight/obesity after adjustment for demographic variables. In addition, several unhealthy dietary behaviors tended to cluster, supporting the importance of evaluating broader behavioral patterns rather than isolated dietary exposures.

The Dietary Risk Score (DRS) demonstrated a progressive association with overweight/obesity prevalence, while the identification of distinct dietary behavioral phenotypes further highlighted the potential contribution of cumulative behavioral burden to weight-related outcomes during emerging adulthood.

Overall, these findings suggest that convenience-oriented eating behaviors may represent important correlates of overweight and obesity among university students. Longitudinal studies are needed to clarify the temporal relationships underlying these associations and to evaluate their implications for nutritional education and university-based health promotion strategies.

## Figures and Tables

**Table 1:** Prevalence of overweight/obesity according to selected sociodemographic characteristics and convenience-oriented dietary behaviors.

Variable	Total (N = 921)	Overweight/Obesity n (%)	*p*-Value	Effect Size
Sociodemographic characteristics				
Women	558	104 (18.6)	<0.001	V = 0.191
Men	363	118 (32.5)		
Age 18–20 years	443	77 (17.4)	<0.001	V = 0.154
Age 21–24 years	478	145 (30.3)		
Rural residence	297	45 (15.2)	<0.001	V = 0.150
Urban residence	624	177 (28.4)		
Convenience-oriented dietary behaviors				
Fast-food <3 times/week	763	162 (21.2)	<0.001	V = 0.148
Fast-food ≥3 times/week	158	60 (38.0)		
Never/sometimes meal replacement with desserts	821	158 (19.2)	<0.001	V = 0.239
Frequent meal replacement with desserts	100	64 (64.0)		
Composite indicator				
Dietary Risk Score (DRS), mean ± SD	1.02 ± 1.03	1.27 ± 1.16 *	0.0017 ^a^	–
Dietary Risk Score (DRS), median [IQR]	1 [0–2]	1 [0–2]	–	–

Data are presented as total frequencies and the number (%) of participants with overweight/obesity within each category. *p*-values were calculated using Pearson’s chi-square test, except for the Dietary Risk Score (DRS), which was compared using the Mann–Whitney U test. Effect sizes are reported as Cramer’s V for categorical variables. DRS, Dietary Risk Score; IQR, interquartile range; SD, standard deviation, * Mean ± standard deviation (SD); ^a^ Mann–Whitney U test.

**Table 2:** Distribution of BMI categories across convenience-oriented dietary behaviors and Dietary Risk Score levels (N = 921).

Variable/ Category	Underweight n (%)	Normal Weight n (%)	Overweight/Obese n (%)	*p*-Value/ Effect Size	*p*-Trend
Fast-food frequency					
Never	4 (10.8)	25 (67.6)	8 (21.6)	<0.001; V = 0.148	<0.001
Rarely	32 (8.8)	276 (76.2)	54 (14.9)		
1–2 times/week	18 (4.9)	246 (67.6)	100 (27.5)		
≥3 times/week	5 (3.2)	93 (58.9)	60 (38.0)		
Fruit and vegetable intake					
<2 times/week	2 (5.9)	26 (76.5)	6 (17.6)	0.808; V = 0.040	0.164
2 times/week	12 (5.9)	149 (73.0)	43 (21.1)		
3–4 times/week	24 (7.0)	235 (68.3)	85 (24.7)		
Daily	21 (6.2)	230 (67.8)	88 (26.0)		
Daily water intake					
<1 L/day	19 (9.0)	151 (71.9)	40 (19.0)	0.072; V = 0.068	–
2 L/day	26 (5.6)	327 (70.6)	110 (23.8)		
>2 L/day	14 (5.6)	162 (65.3)	72 (29.0)		
Meal replacement with desserts					
Never	27 (7.7)	273 (77.8)	51 (14.5)	<0.001; V = 0.239	<0.001
Sometimes	30 (6.4)	333 (70.9)	107 (22.8)		
Frequently	2 (2.0)	34 (34.0)	64 (64.0)		
Dietary Risk Score (DRS)					
0	22 (6.5)	250 (73.7)	67 (19.8)	<0.001; V = 0.147	<0.001
1	20 (6.1)	233 (70.8)	76 (23.1)		
2	13 (8.1)	104 (64.6)	44 (27.3)		
≥3	4 (4.3)	53 (57.6)	35 (38.0)		

Values represent row percentages within each behavioral category. *p*-values were obtained using Pearson’s chi-square test. *p*-trend values derive from linear-by-linear association analyses across ordinal BMI categories and ordinal exposure levels. Effect size is reported as Cramer’s V. BMI, body mass index; DRS, Dietary Risk Score.

**Table 3:** Exploratory sex-stratified multivariable logistic regression analyses for overweight/obesity.

Variable	Women aOR (95% CI)	Men aOR (95% CI)	Interaction *p*-Value
Fast-food ≥3 times/week	2.37 (1.19–4.73)	1.61 (0.84–3.07)	0.284
Frequent meal replacement with desserts	10.10 (4.91–20.75)	7.94 (3.43–18.37)	0.992
Dietary Risk Score (per +1 increment)	0.79 (0.59–1.06)	0.84 (0.62–1.14)	0.764

Exploratory sex-stratified logistic regression models were adjusted for age group and residence environment. Interaction *p*-values were derived from multiplicative interaction terms included in the corresponding multivariable model. aOR, adjusted odds ratio; BMI, body mass index; CI, confidence interval; DRS, Dietary Risk Score.

**Table 4:** Associations between individual convenience-oriented dietary behaviors and overweight/obesity among Romanian university students.

Predictor	aOR	95% CI	*p*-Value
Fast-food frequency			
Rarely (reference)	1.00	–	–
Never	1.52	0.64–3.59	0.342
1–2 times/week	2.19	1.50–3.21	<0.001
≥3 times/week	3.68	2.33–5.79	
Ordinal trend (per category increase)	1.76	1.44–2.17	
Meal replacement with desserts			
Never (reference)	1.00	–	–
Sometimes	1.74	1.21–2.50	0.003
Frequently	10.55	6.40–17.39	<0.001
Linear trend (per exposure level)	2.96	2.30–3.81	
Ordinal logistic regression a	2.08	1.64–2.63	
Daily sweets intake			
Per category increase	1.08	0.92–1.28	0.352
Daily vs. <2 times/week	1.34	0.96–1.87	0.099

All models were adjusted for sex, age group, and residence environment. Ordinal logistic regression model using the four-level BMI classification (underweight, normal weight, overweight, obesity) as outcome. BMI, body mass index; CI, confidence interval; aOR, adjusted odds ratio.

**Table 5:** Multivariable logistic regression model for factors independently associated with normal-weight status among Romanian university students (N = 862; underweight participants excluded).

Predictor	aOR	95% CI	*p*-Value
Daily fruit and vegetable intake	0.83	0.59–1.16	0.269
Adequate water intake (≥2 L/day)	0.81	0.54–1.22	0.319
Fast-food never/rarely (vs. ≥1 time/week)	2.40	1.70–3.39	<0.001
Female sex	1.77	1.28–2.46	
Age 18–20 years	2.25	1.62–3.14	
Rural residence	2.41	1.64–3.53	

Outcome: normal weight (BMI 18.5–24.9 kg/m^2^) versus overweight/obesity (BMI ≥ 25 kg/m^2^). All predictors were entered simultaneously in the multivariable model. BMI, body mass index; CI, confidence interval; aOR, adjusted odds ratio.

**Table 6:** Dietary behavioral phenotypes identified by k-means cluster analysis and their association with BMI status, Dietary Risk Score, and sex distribution (N = 921).

Variable	Convenience-Oriented (n = 283; 30.7%)	Health-Conscious (n = 368; 40.0%)	Low-Water Intake Snacking (n = 270; 29.3%)	*p*-Value
Standardized cluster centers (z-scores)				
Fast-food frequency	0.84	−0.18	−0.63	–
Daily sweets intake	0.60	−0.81	0.48	–
Fruit and vegetable intake	−0.54	0.09	0.43	–
Water intake	−0.17	0.63	−0.68	–
Meal replacement with desserts	0.79	−0.41	−0.27	–
Dietary Risk Score				
Mean ± SD	1.98 ± 1.00	0.35 ± 0.53	0.95 ± 0.77	<0.001 ^a^
BMI categories, n (%)				
Underweight	10 (3.5)	28 (7.6)	21 (7.8)	<0.001 ^b^
Normal weight	181 (64.0)	251 (68.2)	208 (77.0)	
Overweight/obese	92 (32.5)	89 (24.2)	41 (15.2)	
Male sex, n (%)	116 (41.0)	156 (42.4)	91 (33.7)	0.027 ^b^

^a^ Kruskal–Wallis H test for DRS distribution across clusters. ^b^ Pearson’s chi-square test. Cluster centers are presented as standardized z-scores (mean = 0; SD = 1). The three-cluster solution was selected using elbow-method optimization. Hierarchical Ward clustering yielded a similar three-cluster structure. BMI, body mass index; DRS, Dietary Risk Score; SD, standard deviation.

**Table 7:** Final multivariable logistic regression model for overweight/obesity among Romanian university students (N = 921).

Predictor	aOR	95% CI	*p*-Value
Dietary predictors			
Fast-food ≥3 times/week	1.65	1.07–2.52	0.022
Daily sweets intake	1.17	0.80–1.73	0.417
Low fruit and vegetable intake (≤2 times/week)	0.73	0.49–1.09	0.122
Low water intake (<1 L/day)	0.74	0.48–1.13	0.162
Frequent meal replacement with desserts	8.04	4.91–13.18	<0.001
Demographic covariates			
Male sex	2.01	1.43–2.82	<0.001
Age 21–24 years	2.07	1.47–2.93	
Urban residence	3.04	1.99–4.66	

Model performance: Nagelkerke R^2^ = 0.239; Hosmer–Lemeshow *p* = 0.233; apparent AUC = 0.758; optimism-corrected AUC = 0.747. AUC, area under the receiver operating characteristic curve; BMI, body mass index; CI, confidence interval; aOR, adjusted odds ratio.

## Data Availability

The de-identified dataset supporting the conclusions of this article is available from the corresponding author upon reasonable request. The dataset cannot be made publicly available because it contains participant data protected by ethical approval and informed consent.

## Supplementary Materials

The following supporting information can be downloaded at: https://www.mdpi.com/article/10.3390/nu18142368/s1. Supplementary File S1: Questionnaire (English version); Supplementary Table S1. Distribution of nutritional behaviors in the study population and bivariate associations with sex, residence environment, and overweight/obesity (N = 921); Supplementary Table S2. Spearman rank-order correlation matrix (rho) and corresponding *p*-values for the six core dietary behaviors in the study population (N = 921); Supplementary Table S3. Multicollinearity diagnostics (variance inflation factors) for the final multivariable logistic regression model; Supplementary Table S4. Pre-specified sensitivity analyses of the final multivariable logistic regression models; Supplementary Table S5. Sensitivity analyses of the principal dietary predictors of overweight/obesity.

## Abbreviations

The following abbreviations are used in this manuscript:

aOR	Adjusted odds ratio
AUC	Area under the receiver operating characteristic curve
BMI	Body mass index
CI	Confidence interval
DRS	Dietary risk score
FF	Fast-food frequency
FV	Fruit and vegetable intake
OR	Odds ratio
ROC	Receiver operating characteristic
SD	Standard deviation
SWEETS	Daily sweets intake
VIF	Variance inflation factor
WATER	Daily water intake
WHO	World Health Organization
