# Supplementary material for: Convenience-Oriented Dietary Behavioral Patterns Across BMI Classes in University Students: Associations with Overweight and Obesity Risk During the Transition to University Life
Source: Nutrients. 2026 Jul 20;18(14):2368. doi: 10.3390/nu18142368 (PMC13416017; doi:10.3390/nu18142368)
Supplement: Supplementary file 1 [file nutrients-18-02368-s001.zip › File S1. Nutrients_Questionnaire_English.pdf]

Title: Study on Lifestyle, Nutrition, and Health Indicators

Section 1: Participation Agreement (Informed Consent)

Information for participants: You are invited to participate in a research study regarding the correlation between eating habits and biochemical indicators.

- **Privacy:** Participation is anonymous. No names, email addresses (except for Google sign-in process if enabled, but these will not be linked to the responses), contact details or professional identifiers are collected.
- **Data collection:** Collected data are anonymous and do not allow any direct or indirect identification of participants. No names, initials, contact details or other information that could lead to the identification of the person will be collected. All data will be analyzed and reported only in aggregate form. Provided information will be stored securely and confidentially, in compliance with applicable personal data protection legislation (General Data Protection Regulation – GDPR, EU 2016/679). This study is observational in nature and does not involve any additional medical intervention. Completing the questionnaire does not entail medical risks and does not replace specialist medical recommendations or consultations.

- **Data processing:** Responses will be analyzed exclusively in aggregate (group-level). However, please note that theoretically, specific combinations of demographic variables could allow indirect identification.

- **Volunteering:** Participation is voluntary and you may withdraw at any time by closing the form. By completing this questionnaire, you agree that your responses will be used solely for scientific purposes, within academic analyses, doctoral thesis, and scientific articles.

Participants' agreement: By continuing and completing the questionnaire, you confirm that:

- you have read and understood the above information;
- you agree to participate voluntarily in this study;
- you allow the anonymous use of data strictly for scientific purposes.

☐ I agree to participate in this study and to complete the questionnaire.

Question 1 (Mandatory): I confirm that I have read the above information and agree to participate in this study.

[ ] Yes, I agree.

Question 2 (Mandatory): Please enter today's date to validate your consent.
Answer type: Date (DD/MM/YYYY)

Section 2: Demographic and Anthropometric Profile

1. **Gender:**
   [ ] Female
   [ ] Male

2. **Area of residence:**
   [ ] rural
   [ ] urban

3. **Age (range):**
   [ ] 18 - 20 years
   [ ] 21 - 24 years
   [ ] 25- 28 years
   [ ] 29-32 years
   [ ] over 32 years

4. **Height (cm): (ex. 175)**

5. **Current weight (kg):**

6. **Last year's weight (kg):**

Section 3: Eating Habits and Lifestyle

7. **How many times do you eat per day?**
   [ ] 2-3 times
   [ ] 4-5 times

8. **What is your main meal of the day?**
   [ ] Breakfast
   [ ] Lunch
   [ ] Dinner

9. **Do you eat breakfast every morning?**
   [ ] Yes
   [ ] No

10. **Do you believe that breakfast is the most important meal of the day?**
    [ ] Yes

[ ] No

11. Are you a vegetarian?

[ ] Yes
[ ] No

12. What do you usually eat for lunch? (open question)

13. Where do you usally eat lunch?

[ ] cafeteria
[ ] restaurant
[ ] at home

14. What type of food do you usually eat for dinner?

[ ] cooked
[ ] uncooked

15. When do you eat the last meal of the day?

[ ] before 18
[ ] 19-20
[ ] 21-22
[ ] after 22

16. How much water do you drink daily?

[ ] less than 1L
[ ] 2L
[ ] over 2L

17. How many cups of coffee/tea do you drink daily?

[ ] 2 or less
[ ] 3-4
[ ] 5-6

18. What type of food do you usually prefer eating?

[ ] fresh
[ ] frozen

19. How many portions of fruit/vegetables do you eat in a week?

[ ] less than 2 times/week
[ ] 2 times/week
[ ] 3-4 times/week
[ ] daily

20. How many portions of chocolate/candy do you eat in a week?

[ ] less than 2 times/week
[ ] 2 times/week
[ ] 3-4 times/week
[ ] daily

21. What kind of chocolate do you prefer eating?

[ ] dark
[ ] white
[ ] milk chocolate

22. How often do you replace meals with desserts?

[ ] never
[ ] sometimes
[ ] frequently

23. How often do you eat fast food?

[ ] never
[ ] rarely
[ ] 1-2 times/week
[ ] 3 or more times/week

We sincerely thank you for your participation in this study!
Your input is important and will be used to support academic research in the field of nutrition and health.
