# Supplementary material for: Convenience-Oriented Dietary Behavioral Patterns Across BMI Classes in University Students: Associations with Overweight and Obesity Risk During the Transition to University Life
Source: Nutrients. 2026 Jul 20;18(14):2368. doi: 10.3390/nu18142368 (PMC13416017; doi:10.3390/nu18142368)
Supplement: Supplementary file 1 [file nutrients-18-02368-s001.zip › Supplementary Table S1.pdf]

Supplementary Table S1. Distribution of nutritional behaviors in the study population and bivariate associations with sex, residence environment, and overweight/obesity (N = 921).

Behavior / category	n (%)	p-value (Sex)	V (Sex)	p-value (Env.)	V (Env.)	p-value (OW/OB)	V (OW/OB)
Fast-food frequency							
Never	37 (4.0)	0.048 *	0.09	0.054	0.09	< 0.001 *	0.20
Rarely	362 (39.3)						
1-2 times/week	364 (39.5)						
≥3 times/week	158 (17.2)						
Daily water intake							
< 1 L	210 (22.8)	< 0.001 *	0.12	0.119	0.07	0.044 *	0.08
2 L	463 (50.3)						
> 2 L	248 (26.9)						
Candy/chocolate							
< 2 times/week	59 (6.4)	0.109	0.08	0.030 *	0.10	0.225	0.07
2 times/week	343 (37.2)						
3-4 times/week	278 (30.2)						
Daily	241 (26.2)						
Fruits/vegetables							
< 2 times/week	34 (3.7)	0.206	0.07	0.189	0.07	0.475	0.05
2 times/week	204 (22.1)						
3-4 times/week	344 (37.4)						
Daily	339 (36.8)						
Replace meals with desserts							
Never	351 (38.1)	0.737	0.03	0.024 *	0.09	< 0.001 *	0.34
Sometimes	470 (51.0)						
Frequently	100 (10.9)						
Fresh vs frozen food							
Fresh food	830 (90.1)	0.016 *	0.08	0.031 *	0.07	< 0.001 *	0.39

Frozen food	91 (9.9)						
Lunch location							
Home	695 (75.5)	0.099	0.07	0.257	0.05	0.209	0.06
Restaurant	136 (14.8)						
Cafeteria	90 (9.8)						
Dinner preference							
Cooked	802 (87.1)	0.602	0.02	0.854	0.01	0.677	0.01
Uncooked	119 (12.9)						
Tea/coffee (cups/day)							
<=2	754 (81.9)	0.961	0.01	0.507	0.04	0.142	0.07
3-4	106 (11.5)						
5-6	61 (6.6)						
Chocolate preference							
Milk	351 (38.1)	0.758	0.03	0.215	0.06	0.666	0.03
Dark	338 (36.7)						
White	232 (25.2)						
Vegetarian							
No	900 (97.7)	0.726	0.01	1.000	0.00	0.821	0.01
Yes	21 (2.3)						

Values are n (%). p = Pearson chi-square; V = Cramer's V. Sex categories: female vs male. Env. = residence environment (urban vs rural). OW/OB = overweight/obese (BMI ≥ 25 kg/m^2) vs underweight/normal weight. * Statistically significant at $p < 0.05$.
