# Supplementary material for: Convenience-Oriented Dietary Behavioral Patterns Across BMI Classes in University Students: Associations with Overweight and Obesity Risk During the Transition to University Life
Source: Nutrients. 2026 Jul 20;18(14):2368. doi: 10.3390/nu18142368 (PMC13416017; doi:10.3390/nu18142368)
Supplement: Supplementary file 1 [file nutrients-18-02368-s001.zip › Supplementary Table S2.pdf]

**Supplementary Table S2. Spearman rank-order correlation matrix (rho) and corresponding p-values for the six core dietary behaviors in the study population (N = 921).**

| Variable | Fast-food | Daily sweets | Fruits/veg | Water intake | Meal replacement | Frozen-food |
|---|---|---|---|---|---|---|
| **Fast-food** | 1.000 | 0.183 *** | -0.123 *** | 0.046 | 0.206 *** | 0.109 *** |
| **Daily sweets** | 0.183 *** | 1.000 | 0.073 * | -0.145 *** | 0.242 *** | 0.029 |
| **Fruits/veg** | -0.123 *** | 0.073 * | 1.000 | 0.091 ** | -0.129 *** | 0.021 |
| **Water intake** | 0.046 | -0.145 *** | 0.091 ** | 1.000 | -0.055 | 0.006 |
| **Meal replacement** | 0.206 *** | 0.242 *** | -0.129 *** | -0.055 | 1.000 | 0.163 *** |
| **Frozen-food** | 0.109 *** | 0.029 | 0.021 | 0.006 | 0.163 *** | 1.000 |

Spearman rank-order correlation coefficients computed on the 921 students with complete data on all six ordinal/dichotomous behavioural variables. Statistical significance: *** $p < 0.001$; ** $p < 0.01$; * $p < 0.05$. Variables coded as: Fast-food = 0 (Never) - 3 (>= 3 times/week); Daily sweets = 0 (< 2 times/week) - 3 (Daily); Fruits/veg = 0 (< 2 times/week) - 3 (Daily); Water intake = 0 (< 1 L/day) - 2 (> 2 L/day); Meal replacement = 0 (Never) - 2 (Frequently); Frozen-food = dichotomous (Fresh = 0, Frozen = 1).
