# Supplementary material for: Convenience-Oriented Dietary Behavioral Patterns Across BMI Classes in University Students: Associations with Overweight and Obesity Risk During the Transition to University Life
Source: Nutrients. 2026 Jul 20;18(14):2368. doi: 10.3390/nu18142368 (PMC13416017; doi:10.3390/nu18142368)
Supplement: Supplementary file 1 [file nutrients-18-02368-s001.zip › Supplementary Table S3.pdf]

Supplementary Table S3. Multicollinearity diagnostics (variance inflation factors) for the final multivariable logistic regression model.

Predictor	VIF
Dietary predictors	
Fast-food ≥3 times/week	1.089
Daily sweets intake	1.060
Low fruit and vegetable intake (≤2 times/week)	1.033
Low water intake (<1 L/day)	1.041
Frequent meal replacement with desserts	1.108
Frozen-food preference	1.060
Demographic covariates	
Male sex	1.046
Age 21–24 years	1.023
Urban residence	1.037
Summary	
Maximum VIF (excluding constant)	1.108
Interpretation	No multicollinearity

Variance inflation factors (VIFs) were computed for all predictors included in the final multivariable logistic regression model for overweight/obesity presented in Section 3.9. All VIF values were well below conventional thresholds for problematic multicollinearity, indicating absence of substantial collinearity among the simultaneously modelled predictors. VIF, variance inflation factor.
