# Supplementary material for: Convenience-Oriented Dietary Behavioral Patterns Across BMI Classes in University Students: Associations with Overweight and Obesity Risk During the Transition to University Life
Source: Nutrients. 2026 Jul 20;18(14):2368. doi: 10.3390/nu18142368 (PMC13416017; doi:10.3390/nu18142368)
Supplement: Supplementary file 1 [file nutrients-18-02368-s001.zip › Supplementary Table S4.pdf]

Supplementary Table S4. Pre-specified sensitivity analyses of the final multivariable logistic regression model.

Sensitivity analysis	Predictor	OR / β (95% CI)	p-value
**A. Excluding underweight participants (n = 862)**	Fast-food ≥3 times/week	1.97 (1.25-3.12)	0.004
	Frequent meal replacement	7.17 (4.18-12.30)	< 0.001
	Frozen-food preference	18.29 (9.79-34.17)	
	Daily sweets	0.98 (0.64-1.49)	0.909
**B. Outcome restricted to obesity only (BMI >= 30; n_cases = 43)**	Fast-food ≥3 times/week	1.96 (0.92-4.18)	0.081
	Daily sweets	2.54 (1.27-5.07)	0.008
	Low fruit/vegetable (≤ 2 /wk)	0.26 (0.09-0.77)	0.015
	Frequent meal replacement	2.18 (0.93-5.11)	0.074
	Frozen-food preference	1.74 (0.72-4.16)	0.217
**C. Alternative DRS cutoff (DRS >= 2 vs < 2)**	DRS ≥ 2 (binary)	1.90 (1.35-2.66)	< 0.001
**D. Continuous Diff_BMI as outcome (multiple linear regression; R^2 = 0.042)**	Fast-food ≥3 times/week	β = -0.16 (-0.35-0.03)	0.098
	Daily sweets	β = -0.02 (-0.18-0.14)	0.843
	Low fruit/vegetable	β = -0.00 (-0.16-0.16)	0.977
	Low water intake	β = -0.38 (-0.55-0.22)	< 0.001
	Frequent meal replacement	β = 0.21 (-0.02-0.44)	0.079
	Frozen-food preference	β = 0.30 (0.07-0.54)	0.011
	Male sex	β = -0.17 (-0.31-0.03)	0.021
	Urban residence	β = 0.15 (0.00-0.30)	0.048
**E. Ordinal logistic regression (BMI_ord = underweight / normal / overweight / obesity)**	Fast-food ≥3 times/week	β = 0.57 (0.19-0.95)	0.004
	Daily sweets	β = 0.29 (-0.04-0.63)	0.086
	Low fruit/vegetable	β = -0.19 (-0.53-0.14)	0.262
	Low water intake	β = -0.28 (-0.64-0.09)	0.137
	Frequent meal replacement	β = 1.46 (1.02-1.89)	< 0.001
	Frozen-food preference	β = 1.84 (1.39-2.28)	

All sensitivity analyses were adjusted for sex, age group and residence environment. (A) Re-fitting on the subsample with underweight participants excluded confirmed the principal associations. (B) Restricting the outcome to obesity (n_cases = 43) preserved effect directions but reduced precision, with daily sweets and low fruit/vegetable intake

reaching statistical significance specifically against obesity. (C) Dichotomising the DRS at ≥ 2 produced a comparable adjusted OR (1.90), confirming the gradient is not an artefact of category collapsing. (D) Continuous Diff_BMI as outcome confirmed frozen-food preference and urban residence as positive determinants and male sex as inversely associated. (E) The ordinal logistic regression preserved the rank ordering of the principal dietary predictors. Across all five sensitivity analyses, the directionality of the principal dietary associations remained unchanged, supporting the robustness of the central finding.
