# Supplementary material for: Convenience-Oriented Dietary Behavioral Patterns Across BMI Classes in University Students: Associations with Overweight and Obesity Risk During the Transition to University Life
Source: Nutrients. 2026 Jul 20;18(14):2368. doi: 10.3390/nu18142368 (PMC13416017; doi:10.3390/nu18142368)
Supplement: Supplementary file 1 [file nutrients-18-02368-s001.zip › Supplementary Table S5.pdf]

**Supplementary Table S5.** Sensitivity analyses of the principal dietary predictors of overweight/obesity.

| Analysis | Meal replacement with desserts | p-value | Fast-food ≥3 times/week | p-value | Additional findings | p-value |
|---|---|---|---|---|---|---|
| Excluding underweight participants (*n* = 862) | aOR 8.07 (95% CI 4.88–13.35) | <0.001 | aOR 1.64 (95% CI 1.07–2.52) | 0.024 | Daily sweets: aOR 1.10 (95% CI 0.74–1.62) | 0.640 |
| Obesity-only outcome (BMI ≥30 kg/m²; *n* = 43) | aOR 2.36 (95% CI 1.03–5.43) | 0.043 | aOR 1.92 (95% CI 0.91–4.06) | 0.089 | Daily sweets: aOR 2.66 (95% CI 1.34–5.27); low fruit/vegetable intake: aOR 0.25 (95% CI 0.08–0.73) | 0.005; 0.012 |
| Alternative DRS categorization (DRS ≥2 vs. <2) | – | – | – | – | DRS: aOR 1.90 (95% CI 1.35–2.66) | <0.001 |
| Multiple linear regression (Diff_BMI outcome) | β = 0.25 (95% CI 0.03–0.48) | 0.029 | β = −0.17 (95% CI −0.35–0.02) | 0.082 | Urban residence: β = 0.13 (95% CI −0.01–0.28) | 0.074 |
| Ordinal logistic regression (BMI categories) | β = 1.69 (95% CI 1.26–2.11) | <0.001 | β = 0.48 (95% CI 0.11–0.85) | 0.012 | Daily sweets: β = 0.37 (95% CI 0.04–0.70) | 0.028 |

aOR, adjusted odds ratio; BMI, body mass index; CI, confidence interval; DRS, Dietary Risk Score.
